# Lifestyle and fertility: the influence of stress and quality of life on male fertility

**DOI:** 10.1186/s12958-018-0436-9

**Published:** 2018-11-26

**Authors:** Alessandro Ilacqua, Giulia Izzo, Gian Pietro Emerenziani, Carlo Baldari, Antonio Aversa

**Affiliations:** 1 Department of Movement, Human and Health Sciences, Section of Health Sciences, University of Rome “Foro Italico”, Rome, Italy; 2 Department of Experimental and Clinical Medicine, Magna Graecia University of Catanzaro, Catanzaro, Italy; 3 University eCampus, Novedrate, Italy

**Keywords:** Male fertility, Lifestyle factors, Stress, Nutrition, Physical exercise, Natality

## Abstract

**Background:**

Male infertility is a widespread condition among couples. In about 50% of cases, couple infertility is attributable to the male partner, mainly due to a failure in spermatogenesis. In recent times, the crucial role that modifiable lifestyle factors play in the development of infertility have generated a growing interest in this field of study, i.e. aging, psychological stress, nutrition, physical activity, caffeine, high scrotal temperature, hot water, mobile telephone use. Several studies have investigated associations between semen quality and the presence of lifestyle stressors i.e. occupational, life events (war, earthquake, etc.) or couple infertility; overall, these studies provide evidence that semen quality is impaired by psychological stress. In this review, we will discuss the impact of quality of life (modifiable lifestyle factors) and psychological stress on male fertility. In addition, the role that increased scrotal temperature along with inappropriate nutritional and physical exercise attitudes exert on male fertility will be presented.

**Conclusion:**

The decline of male fertility, particularly associated with advancing age, incorrect lifestyles and environmental factors plays an important role on natality, and its consequences on the future on human population makes this an important public health issue in this century. Thus, modification of lifestyle through a structured program of educational, environmental, nutritional/physical exercise and psychological support, combined with the use of nutraceutical antioxidants can prevent infertility and therefore, may help couples to obtain better quality of life and improved possibility to conceive spontaneously or optimize their chances of conception.

## Background

In industrialized nations, decreasing the number of people affected by infertility has become a top priority for many health organizations. In Europe, several studies have suggested a possible decline in fecundity of the population [1]. The net effect has been a 7% decrease in fecundability, as suggested by several studies [2]. If the trend observed over 15 years will be extended to 45 years, the reduction in fecundability would be doubled and finally could increase to the extreme hypothesis of a 50% reduction [3]. Infertility affects both men and women. In 50% of involuntarily childless couples, a male-infertility-associated factor is found together with abnormal semen parameters. A fertile partner may compensate for the fertility problem of the man and thus infertility usually manifests itself if both partners have reduced fertility [4].

Lifestyle factors can be modified to enhance overall wellbeing and they are ultimately under one’s own control. Reproductive health can be affected positively or negatively by multiple factors, i.e. age of paternity, nutrients, physical exercise, obesity, caffeine, scrotal temperature, clothing, hot water, mobile telephones [5] that can thus impact the quality of life of sperm parameters and DNA damage induced by reactive oxygen species (ROS) [6]. Also, the altered balance between antioxidant system [7] and oxidative stress, may determine poor fertilization/ embryonic development, pregnancy loss, birth defects and childhood cancer [8–10]. In this review we will present evidence that modification of lifestyle through a structured program of educational, environmental, nutritional/physical exercise and psychological support, combined with the use of nutraceutical antioxidants can prevent infertility and therefore, may help couples to obtain better quality of life and improved possibility to conceive spontaneously or optimize their chances of conception.

### Role of stress on male fertility

Stress is a prominent part of any society and infertility itself is stressful, due to social pressures, testing, diagnosis, treatments, failures, unfulfilled desires and even economic costs with which it is associated [11]. Semen parameters may be potentially linked to stress, whose presence may reduce luteinizing hormone (LH) and testosterone pulsing, thus reducing in turn spermatogenesis and sperm quality [12, 13].

Pre-clinical data have shown that *acute* stress might impair testicular function; testicular tissue from stressed rats shows higher levels of cortisol displayed apoptosis of both germ cells and Leydig cells [14, 15]. By contrast, the net effects of stress might be determined by *chronic* as demonstrated by the presence of glucocorticoid receptors (GRs) in Leydig, [16], Sertoli [17] and germs cells [15]; permanently high levels of glucocorticoid are believed to induce apoptosis of all cell types [15–17]. The Leydig cell is the primary target of glucocorticoid regulation in the testes. Today, our current understanding of glucocorticoid signaling in the context of reproductive physiology is limited. In humans, stress results in a variety of neuroendocrine, immune and behavioral responses. Recently, new evidence supporting the GR response to glucorticoid in the regulation of Sertoli and Leydig cell’s function has been suggested for a single nucleotide NR3C1 polymorphisms (BcII [rs41423247] [18]. Thus, this variant gene (in an over-dominant manner with heterozygotes) is strongly associated with better sperm motility and a better testicular function [18].

In humans, polymorphism of the GR could suggest a response variability to stress [19]. An isolated stress such as a job, life events, and even social strain or two simultaneous stressful life events may have a significant negative impact on sperm quality [11]. The perceived stress of providing a semen sample was reported to be negatively linked to overall semen quality with a 39% decrease in sperm concentration, 48% decrease in motility, and worse overall semen parameters on the day of oocyte retrieval, although there was no change in either volume or morphology [20, 21]. Futhermore, environmental disasters, war or “stressful life events” are major determinants that do not allow to quantify their impact on fertility, thus determining underestimation of the actual stress burden. A high stress level may occur owing to a continuous high stress in daily life without occurrence of specific stressful exposures. This might explain the uncertain results, and a study setup based on stress due to environmental disasters or war should be preferably accompanied by an assessment of perceived stress [12, 22–26].

Many studies show that men undergoing infertility treatment [27–29] or men from the general population [30] have a decline in semen parameters during infertility treatment, but it is difficult to distinguish whether stress is a cause or a consequence of decreased semen quality in such studies. Stress can increase after diagnosis of male infertility, follow-up appointments, and failed in vitro fertilization treatments [11]. Men undergoing infertility treatment met the criteria for having an anxiety disorder or depression, the latter being more common [12]. Coping with various lifestyles may also affect fertility. It was reported that actively coping with stress, such as being assertive or confrontational, may negatively impact on fertility, by increasing adrenergic activation, leading to more vasoconstriction in the testes [31]. This vasoconstriction results in a lower testosterone level and decreased spermatogenesis. While men are not often thought to report their anxiety or sexual stress, the link between anxiety and sexual stress was surprisingly strong [32]. Two studies investigating self-reported “daily-life-stress” in men from the general population, while controlling for relevant confounders, have shown controversial results on semen parameters. One study detected linear negative associations between perceived stress and sperm motility, sperm concentration, and percentage of morphologically normal spermatozoa [33]. The other study did not find any association between stress and semen parameters, but found that fecundability decreased with increasing stress score in men with low semen quality [34]. Thus, a negative association between self-reported stress and semen quality reported in many studies represents a public health concern (Table 1). Psychological stress might be a modifiable or reversible factor, which is important in a clinical setting [5]. Future studies should objectively assess the impact of stress and prospectively evaluate whether timely counseling aimed at lowering stress levels may restore semen quality, and attempt to clarify the underlying biological mechanisms by which stress affects semen quality.

**Table 1:** Effects of psychological stress on semen parameters: clinical studies

First author and year	Population	Country	Stress exposure and/ or assessment	Semen parameter affected	Adjustment	Study design
Stress due to environmental disasters						
Fukuda 1996 [24]	27 infertile men	Japan	Earthquake	↓Motility →Sperm conc	Unadjusted	Longitudinal
Abu-Musa 2007 [22]	10,000 semen samples	Lebanon	War	↓Sperm conc [Morphology →Volume, motility	Unadjusted	Cross- sectional
Stress due to examinations or other stressful life events						
Hjollund 2004 [34]	418 men	Denmark	Self-rated stress	→Sperm conc, semen volume, total count, morphology, motility	Age, smoking, alcohol, caffeine, reproductive disorders, BMI,	Longitudinal
Eskiocak 2005 [23]	34 students	Turkey	University examinations	↓Motility, sperm conc, morphology→ Semen volume	Unadjusted	Longitudinal
Zorn 2008 [32]	1076 infertile men	Slovenia	Life events	Sperm conc, motility, morphology	Age, smoking, abstinence time, cryptorchidism, varicocele	Cross-sectional
Gollenberg 2010 [12]	744 fertile men	USA	Life events	↓Sperm conc, total count →Morphology, motility	Center, age, race, education, fever, abstinence time	Cross-sectional
Nordkap L 2016 [30]	1215 young men	Denmark	self-reported stress	↓ sperm count,motility and morphology ↑ FSH→	Age, reproductive disorders, alcohol, BMI, caffeine, cannabis, stress	Cross- sectional
Occupational stress						
Hjollund 2004 [34]	399 men	Denmark	Work-related	→Sperm conc, volume, total count, morphology	Age, reproductive disorders, alcohol, BMI, caffeine	Cross-sectional
Janevic T 2014 [33]	327 infertile men	Poland	Work-related Self-rated stress	Morphology	Age, reproductive diseases, alcohol, BMI, smoking, duration of infertility	Cross-sectional
Stress due to infertility						
Pook 2005 [31]	120 infertile men	Germany	Self-rated stress	↓ Sperm conc	Unadjusted	Longitudinal
Vellani 2013 [28]	94 male IVF-patients / 85 controls	Italy	Self-rated stress	↓Motility, sperm conc,total count, volume	Unadjusted (but excluded men with diseases)	Cross- sectional
Bhongade 2014 [27]	70 infertile men	India	Self-rated stress	↓Sperm conc, motility, morphology	Age, abstinence time	Cross- sectional

### Quality of life and male fertility

Currently, increased life expectancy, advanced age of marriage, various socio-economic factors and an overall change in role of women in society has led couples to start their family at a later age. The increased accessibility to assisted reproductive techniques (ART) has increased the chance of older parents with poor pregnancy outcomes to conceive children, hence, increasing the average paternal age at first childbirth. Increased paternal age is a major determinant fo testicular function [35, 36], reproductive hormones [37], sperm parameters [38, 39], sperm DNA integrity [40], telomere length [41], de novo mutation rate [42], chromosomal structure [43] and epigenetic factors [44, 45]. These changes negatively affect fertility and reproductive outcomes in older couples, contributing to higher incidences of congenital birth defects [46] and fetal deaths [47]. Increasing male age has also been shown to be associated with numerous disorders like achondroplasia [48], autism [49], schizophrenia and bipolar disorders [45]. Male aging results in the loss of antioxidant activity and elevated levels of ROS [50]. This imbalance between ROS and antioxidants causes oxidative stress and is well documented in the male reproductive tract [51, 52] and in the spermatozoa of aging rodents [53]. If not maintained within normal physiological levels, ROS can damage cellular macromolecules, inducing stress signaling and, at high levels, cell death [54]. A recent study confirmed that aging reduces fertility and the numbers of Sertoli and germ cells in mice with complete absence of either catalase (CAT-null (Cat−/−) or superoxide dismutase 1 (SOD1-null (Sod−/−) [55]. Thus, suggesting that these enzymes appear critical to the maintenance of germ cell quality with aging.

In humans, although spermatozoa are continuously produced with advanced paternal age, there is a growing body of evidence indicating that advanced paternal age is associated with negative impact on the quality of male germ cells [56], the number of Sertoli cells [57] and the number of Leydig cells [58]. A negative association between increasing paternal age and testicular volume was noted by several studies [35, 59]. In a study it was found that compared to the age group 18–40 years, men aged > 75 years had 31% smaller mean testicular volume [59]. In addition, some authors reported the thickening of basal membrane of seminiferous tubules with age [35] as well as disturbances in blood supply in senile testes have been associated with negative changes in spermiogenesis and thickness of basement membrane [60].

Male aging is characterized by different changes in the endocrine function. Hormonal changes are characterized mainly by a reduction of the biosynthesis of testicular inhibin B by Sertoli cells with increased secretion of follicle stimulating hormone (FSH) [61]. Leydig cells are responsible for testosterone production. The number of Leydig cells tends to reduce with increasing paternal age [58]. The average total number of Leydig cell nuclei decreases by half in age group of 50–76 years compared to age group of 20–48 years [58]. Wu et al. reported that age-affected testicular atrophy is a result of Hypothalamic-Pituitary-Testicular Axis alterations that disturb the functions of various reproductive hormones [62].

In a study where semen values of men above 45 years of age were analyzed, four measured parameters (semen volume, sperm concentration, sperm motility, and sperm morphology) and one derived parameter (total sperm count) were calculated according to the age range, and these values were compared to the reference values of the World Health Organization [63]. After the age of 45, semen volume gradually decreases due to functional decline of accessory glands [63]. In addition, sperm morphology is also affected with aging and the percentage of sperms with normal morphology begins to decrease after the age of 40 [64]. The age of a man is directly related to increase of sperm DNA fragmentation, due to elevation of oxidatixe stress [65]. Oxidative stress due to increased production of ROS or reduced antioxidant reserves, is responsible for a majority of DNA fragmentations (almost 80%) occurring during infections, inflammation or in cases of various clinical diagnosis of male infertility [66]. Recently, a meta-analysis confirmed that paternal aging led to a decrease in sperm parameters except for sperm concentration; however, impaired DNA fragmentation and reduced progressive motility were suggested as diagnostic parameters to be considered during fertility treatment of older men [67].

Also, as already noted, advanced paternal age increases sperm DNA fragmentation and may negatively affect the IVF/ICSI success rates [68, 69]. Despite increasing evidence of positive correlation between sperm DNA fragmentation and reduced male fertility, current guidelines do not support the routine use of sperm DNA integrity assessment in clinical practice [70]. Thus, it is clear that advanced paternal age should be considered as a risk factor for possible genetic disorders of newborns and we recommend to use caution in counselling couples with advanced age wanting to conceive with ART because of this evidence.

### Antioxidants

A new emerging role in the male infertile management is the use of antioxidants [71]. They are molecules such as albumin, ceruloplasmin, and ferritin; and an array of small molecules, including ascorbic acid, α-tocopherol, β-carotene, reduced glutathione, uric acid, and bilirubin or enzymes superoxide dismutase, catalase, and glutathione peroxidase [71]. They help to remove ROS excess in the seminal ejaculate and assist in the conversion of ROS to compounds that are less detrimental to cells [71]. If there is abundancy of ROS than the local antioxidants can remove, it results in increased oxidative stress thus impairing sperm protein, lipid and DNA damage and sperm dysfunction [71]. The ascorbic acid (vitamin C) is a known antioxidant present in the testes with the precise role of protecting the latter from the oxidative damage [72]. It also contributes to the support of spermatogenesis, at least in part through its capacity to maintain this antioxidant in an active state [72]. Vitamin C is itself maintained in a reduced state by a GSH-dependent dehydroascorbate reductase, which is abundant in the testes [72]. An emerging role is attributed to myo-inositol, a precursor of the second messenger Ins (1,4,5) P3 [73, 74]. It modulates specific protein phosphorylation process and intracellular Ca++ concentration through one sperm-specific Ca++ − permeable channel (CatSper) in the plasma membrane of the flagellar principal piece, hence it may be beneficial to sperm motility [75–79]. Another scavenger, N-Acetyl cysteine (NAC), is an amino acid that may exhibit antioxidant properties after being converted into cysteine, which is a precursor of glutathione [80]. In vitro studies have demonstrated a beneficial role for NAC on germ cell survival [81] through reduction of ROS levels, thus improving sperm motility [82]. However, most clinical studies using any antioxidant produced controversial results. A double-blind, placebo controlled, randomized study investigated the effect of a log-term administration of selenium and N-acetyl-cysteine on 468 infertile men with idiopathic oligo-asthenoteratospermia suggesting a beneficial effect [83]. Despite a positive association between vitamin D levels and semen quality (sperm motility), there is no proof-of-fact that its administration is able to improve sperm parameters [84]. A Cochrane meta-analysis of 33 trials, suggested that men who use oral antioxidants had a slightly significant increase in live birth rate when compared to controls [85]. Subfertile males using antioxidants, may improve live birth rates for couples attending fertility clinics [85]. Currently, we can conclude that there is no indication neither for screening infertile patients for ROS generation or seminal oxidative stress or treating them with specific antioxidants in the clinical setting once diagnostic workup is concluded in favour of a specific inflammatory etiology.

### Nutritional factors

Nutritional factors are known to be critical determinants of normal reproductive function in both sexes [86]. A combination of reduced physical exercise, changes in dietary composition and increased energy intake have contributed to a growing worldwide epidemic in obesity [87, 88] and diabetes [89], with serious impacts on several aspects of health, including reproductive system health [88, 90]. Moreover, there is increasing evidence indicating a direct relationship between incorrect nutritional attitudes in decreased sperm quality.

Recent evidences from both animal and human studies indicate that *high fat diets* result in impaired reproduction, by affecting molecular and physical structure of sperm as well as the health of the developing fetus and subsequent offspring [90, 91]. The exposure to a *high fat diet* during that period leads to long-term changes in the reproductive system and metabolism of male rats, so it may implicate reproductive and metabolic programming mechanisms [92]: a reduction in seminiferous epithelium height and seminiferous tubular diameter [93], reduced sperm concentration, viability, motility and DNA integrity [94]. On the other hand, adult male Wistar rat offspring born to obese mothers after a long term of regular voluntary physical activity and diet leads to a reduction of adipose tissue and an improved sperm quality and fertility [95]. These beneficial effects were associated to decreased testicular oxidative stress biomarkers and increased sperm antioxidant activity found in exercised animals [95]. Rato et al. reported that testicular physiology is sensitive to alterations of whole-body metabolism and that testicular metabolism can be disturbed by *high-energy diet intake*, such as trans fatty acids and saturated fats and obesity [96]; other authors suggested that chronic inflammation can provoke an impairment of sperm concentration and motility [97].

Emerging data suggest the role of an individualized diet in order to improve semen parameters. It should be characterized by high intakes of *fruits and vegetables* [98, 99], *legumes* [98] and *fish* [100–102], possibly as sources of antioxidants and polyunsaturated fatty acids (among which omega-3) and negatively associated with diets including meats (processed meat in particular) and full-fat dairy products that are sources of saturated fats [103]. In general, fruit and vegetable intake showed a consistently positive association with better motility and morphology [98, 99]. According to the Mediterranean diet score, a high adherence to this diet is strongly associated to better sperm parameter i.e. count, motility and morphology [104], and a lower DNA fragmentation index [105] than those people with lower adherence. By contrast it is known that the frequent use of red meat is negatively associated with sperm parameters [106].

The abuse of high caffeine-content energy drinks has increased in recent years. 28% of children and 31% of adolescents are reported to be regular consumers and this has been hypothesized to influence semen parameters [107]. As suggested by animal studies [108, 109], caffeine easily crosses biologic membranes and is rapidly distributed throughout the body and has been found in saliva, breast milk, the embryo and the fetal rat testis [110]. In humans, prenatal caffeine exposure impairs male gonadal development and thus later gonadal function [111]. However, the mechanism behind the possible harmful effect of caffeine is not well clarified. Coffee consumption has been hypothesized to influence not only semen parameters, but also sperm DNA integrity. Caffeine intake, possibly though sperm DNA damage, may negatively affect the male reproductive function [112, 113]. However, we can conclude that there is no clear association between caffeine and fertility indexes, so this relationship remains unclear and, in some ways, contrasting. It is our opinion that all the quoted observational studies regarding these nutritional factors have proven associations but not causations, the associations need to be confirmed with larger prospective cohort studies and especially with well-designed randomized controlled trials.

### Physical exercise

The beneficial effects of a correct physical exercise on cardio-metabolic parameters are well known [114–116]. Animal studies support the evidence that impaired sperm quality and fertility potential in rat offspring from obese dams, can be ameliorated by exercise performed during adulthood [95]. In mice, a low intensity swimming training improves reproductive system without affecting adiposity in obese animals, which suggests that adiposity itself is not the sole determinant in the impaired sperm function [90]. So, animals exposed to high fat diet and physical exercise, show an attenuation of fat visceral deposits, which can be associated with protection of reproductive system [117]. By contrast, there are conflicting data on the effect of physical activity (PA) on male fertility in humans. Observational studies conducted on general populations and student populations do not provide evidence of any improvement of semen parameters by PA [118]. Initial studies demonstrated that during continuous strenuous exercise, semen parameters and testicular function can be affected negatively by testicular heating [119], oxidative stress (ROS formation) [120], DNA fragmentation [121] and gonadotropin suppression [122].

Physically active subjects have been reported to have higher numbers of motile spermatozoa with normal morphology than sedentary controls [123, 124] and an improvement of sperm parameters has been found after reducing the exposure time of tv-watching [125]. Recent studies suggest that moderate-intensity continuous training may be more advantageous on the oxidant/antioxidant markers in seminal plasma than high-intensity continuous training and high-intensity interval training [126]. Finally, it is worth remembering that many evidences support the fact that continuous bicycling exerts a negative correlation with both total motile sperm counts and sperm concentration because of its influence on scrotal temperature [127]. We can conclude that any kind of extreme or agonistic physical activity may expose subjects to an increased risk of worsening in the reproductive function; the withdrawal of these activities as well as the recommendation of a supervised physical activity may improve fertility especially in patients with concomitant comorbidities i.e. diabetes/obesity.

### Temperature

The exposure of testes to an increase of temperature can impair fertility through the alteration of sperm parameters (number, motility and morphology) and the damage of sperm membrane integrity [128–131]. The temperature of the scrotal sac reflects testicular temperature and its thermoregulation is fundamental defensive mechanisms [132]. Higher temperatures promote increased ROS generartion with subsequent damage on the sperm plasma membrane and determinate DNA fragmentation of both nuclear and mitochondrial genomes, conducing to cell damage and apoptosis [133]. Animal studies support the concept that elevated testicular temperature by 1–1.5 °C resulted in reduction of the testes size, lower sperm production, abnormal forms [134] and lower motility [135–137]. Heat stress can affects testes especially cells with high mitotic rate, like mature spermatozoa spermatocytes and spermatids [130] According to studies conducted on mice, hyperthermia affects sperm cells determining DNA damage and apoptosis by intrinsic or extrinsic pathway [138, 139]. The consequence is a poor fertility capacity in vivo and in vitro.

Clinical studies suggest that slight variations of the testicular temperature may bring to alterations of spermatogenesis according to the delicate temperature sensitivity of testicular DNA synthesis, with temperature maximal sensitivity at 31 °C, whereas for RNA and protein synthesis the maximal sensitivity is 37–40 °C [132]. An increase of 1 °C is correlated to a 14% drop in the spermatogenesis with poorer sperm production [132]. Studies have found that high temperature exposure of sperm led to increase in apoptosis [132]. Accordingly, also portable computers seem to have thermal and non-thermal effects on male fertility, but data in literature are poor and inconclusive [140]. Non -thermal effects are attributed to radiofrequency exposure that can cause a decrease in sperm motility and morphology [141], while thermal effects are more possibly causing detrimental effects. To this end, there is increasing concern that the use of mobile phones, a source of low-level radiofrequency electromagnetic fields (RF-EMF), may be associated with decreased semen quality [142]. There are also some experimental evidences in rats that exposures to mobile phone RF-EMF may lead to histological changes to the testes, disrupted spermatogenesis, and increases in rectal temperature, but, again, the results are also conflicting [143, 144]. In laptop users, both thermal and non-thermal mechanisms have been similarly involved [145]. The state of the art on this topic is limited and is in progress. Prolonged sitting in the car is another risk factor for the rise of testicular temperature, that increases of about 2 °C after 2 h of sitting [146]. Finally, we want to highlight that the type of clothing a man chooses to wear, may have effects on reproductive health i.e. tight fitting underwear and pants showed a relative risk of 2.5 of having impaired semen quality [147]. Thus, it has been suggested that tight fitting versus loose fitting underwear is detrimental on sperm parameters. In accordance, also hot baths, jacuzzis, or saunas may also worsen fertility parameters [148]. A new chance to improve sperm parameters could be scrotal cooling. A randomized controlled trial on scrotal cooling using a hydrogel pad is in the initial recruitment phase [149]. Systematic review aimed to demonstrate beneficial effect of scrotal cooling on male fertility failed to demonstrate real efficacy in pregnancy rates [150]. We can conclude that increased temperature of the scrotal sac may represent an increased risk factor for all men in reproductive age; we recommend that any man seeking fertility is aware of such risk and also recommend the prevention of such risk factor in young, not-father men who are exposed.

## Conclusions

**Fig. 1:**
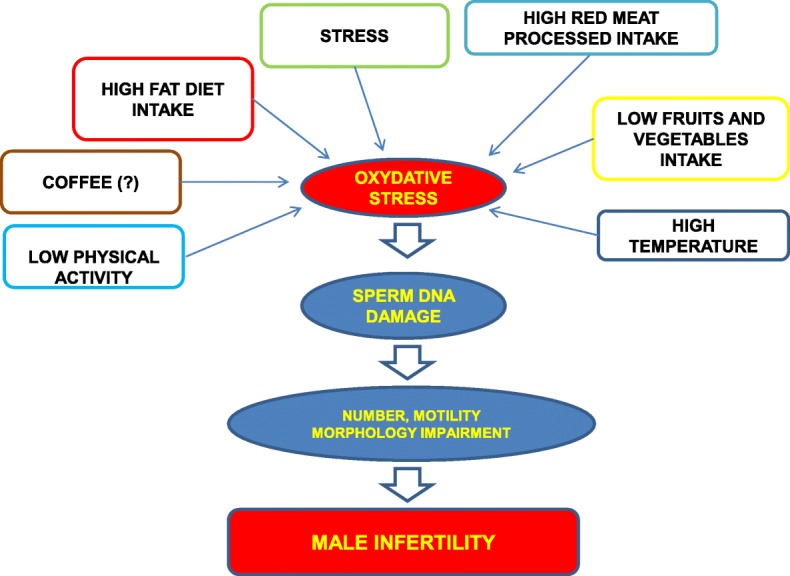
Schematic representation of different effect of incorrect lifestyle factors on male fertility

The crucial role that modifiable lifestyle factors play in the development of male infertility has generated a growing interest in this field **(**Fig. 1**)**. There are associations between psychological attitudes and infertility, but at present it is hard to establish a cause-effect relationship. While stress (physical, emotional, biological, etc) can reduce the potential of male fertility, there is no general consensus on how to measure it objectively. We feel confortable in recommending a special consideration for both partners’age, to thoroughly increase the odds of having a successful pregnancy and to avoid the risk for possible genetic disorders of newborns. We recommend using caution in counselling couples with advanced age wanting to conceive with ART because of this evidence; they should be timely reassured in order to reduce the exposure to stressors. A number of studies have confirmed a beneficial effect for antioxidants in reversing oxidative stress-induced sperm dysfunction specifically in patients with idiopathic male infertility. Their use should be thought of, but not before a diagnostic workup because a robust evidence on this topic is an arduous process. Inappropriate eating behaviors i.e. consuming high fat diet and sedentary lifestyle have been investigated by pre-clinical and clinical studies, so they seemed to worsen sperm parameters. Appropriate nutrients and eventual weight loss may positively influence male fertility. Indeed, it is a fact that obese and overweight men are also predisposed to develop hormonal dysfunctions that may impair their infertility. Up to now, data about choosing supplements or food groups are available only from cross-sectional or case-control studies and no conclusive data about them, i.e. caffeine, are available. PA may positively influence male fertility. A supervised PA administered by a specialist is recommended in any infertile subject with concomitant comorbidities i.e. diabetes/obesity, in order to attain an improvement of fertility, while exercising heavily should be avoided. Today, even if there are suggestive data on the possible influence of other factors such as the type of underwear or clothing or mobile phones use or hot water, little is known about the actual evidence, linking cessation of exposure of lifestyle modifiable factors with resumption of fertility. An access to an andrologist should be encouraged in order to obtain lifestyle recommendations combined with the possible use of nutraceutical antioxidants. The correction of inappropriate lifestyles could improve the degree of DNA fragmentation (even if these latter tests are not recommended in the routine screening) and facilitate the prevision of better quality of life to couples attempting to improve fertility chances of success and minimizing the need for costly and invasive infertility treatment.

It is our opinion that all the quoted observational studies regarding these nutritional factors may prove associations but not causations. Properly-designed randomized controlled trials are needed to conferm these correlations. Also, the effects of reducing/removing the exposition is not applicable to human studies for ethical reasons.
